# Heavy Metals Risk Assessment and Source Apportionment in Agricultural Soils of the Central Yunnan Dry-Hot Valley

**DOI:** 10.3390/toxics14050366

**Published:** 2026-04-24

**Authors:** Lin Song, Tao Zhang, Hedian Yan, Jie Xu, Weizhi Chen, Yong Ba, Hu Wang, Kun Qian, Yuanlong Li, Wenlin Wu, Ya Zhang

**Affiliations:** 1 Kunming General Survey of Natural Resources Center, China Geological Survey, Kunming 650100, China; 18288271618@163.com (L.S.); 18687131557@163.com (J.X.); cwz_email@163.com (W.C.); 17787405118@163.com (Y.B.); cy2628554875@163.com (H.W.); 13700636184@163.com (K.Q.); 18787304302@163.com (Y.L.); 18287183791@163.com (W.W.); 2 Innovation Base for Eco-Geological Evolution, Protection and Restoration of Southwest Mountainous Areas, Geological Society of China, Kunming 650100, China; 3 College of Resources and Environment, Yunnan Agricultural University, Kunming 650201, China; envzhangtao@163.com (T.Z.); yanhedian2023@163.com (H.Y.)

**Keywords:** farmland, heavy metals, source apportionment, APCS-MLR, PMF, Random Forest

## Abstract

Heavy metal contamination in agricultural soils threatens ecosystem safety and sustainable land use, particularly in geologically sensitive areas. This study aimed to assess the pollution status, ecological risks and source contributions of eight heavy metals (Hg, Cd, Pb, As, Cr, Cu, Ni and Zn) in soils from a dry-hot agricultural region of central Yunnan, China. To improve source apportionment, this study applied and compared three models: APCS-MLR, PMF, and Random Forest. Analysis of 1790 soil samples showed mean concentrations (mg/kg) of 0.03 for Hg, 0.17 for Cd, 25.01 for Pb, 7.46 for As, 85.91 for Cr, 36.20 for Cu, 31.75 for Ni, and 69.24 for Zn. Pollution assessment indicated that Cu and Cd were the main pollutants, while ecological risk assessment identified Cd and Hg as the dominant ecological risk factors. Four major sources were identified: industrial hybrid sources, natural background, atmospheric deposition and agricultural activities, with industrial hybrid sources contributing the largest share. These results indicate that integrating APCS-MLR, PMF, and Random Forest provides a more reliable framework for source identification and supports targeted soil pollution control in regions affected by both natural and anthropogenic inputs.

## 1. Introduction

Soil, as the foundation for the growth of terrestrial plants on the Earth’s surface, is an important part of the Earth’s ecosystem. It participates in coordinating the atmosphere, lithosphere, hydrosphere, and biosphere, and is closely related to human survival [1]. At present, soil pollution problems of varying degrees exist in countries around the world [2]. In particular, heavy metal pollution has become a global focus [3,4]. According to the National Soil Survey Bulletin, Heavy metals such as mercury (Hg), cadmium (Cd), arsenic (As), lead (Pb), and chromium (Cr) in Chinese soil are the main elements exceeding the standard, which pose an increasing threat to agricultural production [5]. Although zinc, iron, and other elements can promote plant growth to a certain extent [6,7,8], most of the heavy metals exceeding the standard in the soil have strong biological toxicity, which will greatly affect soil fertility and microbial activity in the long term, leading to the decline of crop yield and quality [9]. Meanwhile, due to the heavy metals’ high persistence and mobility in the environment, they can enter organisms through the food chain, accumulate and amplify, thus directly threatening human health [10].

The heavy metals in the soil accumulate under the action of both artificial and natural factors. Natural factors are mainly related to geological movements, while human activities include industrial emissions, agricultural fertilization, vehicle exhaust, etc. [11,12,13]. These factors together affect the accumulation and diffusion of heavy metals in soil, forming a complex pollution chain. For long-term cultivated land, although the content of soil organic matter has increased, its heavy metal content and migration will also increase with time, further increasing its ecological risk [14]. For the karst landform area in southwest China, its special geological structure makes the migration of heavy metals in soil stronger than other regions [15]. Yunnan Province is located in this area and is relatively fragile in ecology due to the influence of geological causes and climate factors [16]. At the same time, Yunnan province is located in the upper reaches of the Yangtze River Basin, and its soil quality directly affects the ecological security of the whole basin [17,18]. Therefore, performing risk assessment and source apportionment of heavy metals in the soils of typical agricultural zones in central Yunnan Province not only facilitates the revelation of the current soil quality status in this region, but also furnishes critical foundations for formulating scientifically rigorous and practically effective prevention and control strategies. This endeavor is of paramount significance for safeguarding agricultural production safety and maintaining ecological environmental health.

The methods of geological statistics and multivariate statistical analysis can be used to evaluate the soil quality and potential ecological risk assessment in the study area [19]. The absolute principal component linear regression model is a simple calculation method for source apportionment based on principal component scores [20]. The Positive Matrix Factorization (PMF) model is a mature source–receptor model proposed by the U.S. Environmental Protection Agency (U.S.EPA). It can accommodate datasets containing missing values and discrete values [21]. These two receptor models have emerged as pivotal methodologies for environmental pollution source attribution, leveraging their capabilities in identifying contamination sources and quantifying their contribution proportions [22,23]. It is widely applied to the source apportionment of pollutants in environmental media such as the atmosphere, water, and soil. Existing research shows that it has high accuracy and reliability, and can effectively identify the sources of pollutants in the environment and quantify their contribution rates [24,25,26,27]. However, these two models exhibit inherent limitations in addressing complex pollution source scenarios, particularly regarding their suboptimal analytical performance for specific elements [28]. The Random Forest model is a machine learning algorithm based on the binary split of variables for prediction, which has the characteristics of simplicity and efficiency [29]. It is widely used to predict the impact of various factors on the content of environmental pollutants and exhibits good accuracy and stability [30,31,32]. For the karst landscape area in southwestern China, some studies have used Enrichment Factor, Geoaccumulation Index, and Potential Ecological Risk Index to conduct environmental pollution assessments and pollution source apportionment for abandoned metal mines, special crop production areas, and typical watersheds, with results demonstrating good applicability [33,34,35]. However, there are widespread regional limitations, small sample sizes, and the use of a single model for analysis. By combining the advantages of multiple models and comprehensively analyzing the main factors affecting the soil heavy metal content in the region, the sources of heavy metals can be identified more accurately, and their environmental and ecological risks can be evaluated.

The primary goal of this study is to comprehensively assess heavy metal contamination in soils of typical agricultural areas in central Yunnan. To achieve this goal, the study focuses on the following specific objectives: (1) To investigate the concentrations and spatial distribution characteristics of eight heavy metals (Hg, Cd, Pb, As, Cr, Cu, Ni, Zn) in soils within typical agricultural areas of central Yunnan. (2) To analyze soil quality and environmental risk levels in the study area based on assessment methods including the Enrichment Factor (EF), Geo-accumulation Index (Igeo), Single Factor Pollution Index (Pi), Nemerow Integrated Pollution Index (PN), individual heavy metal Environmental Risk Index (Ei), and Potential Ecological Risk Index (RI). (3) To identify the influencing factors, primary sources and contribution rates of heavy metals in farmland soils through multivariate statistical analysis, Absolute Principal Component Score-Linear Regression (APCS-MLR), Positive Definite Matrix Factorization (PMF), and Random Forest (RF) analysis.

## 2. Materials and Methods

### 2.1. Study Area Establishment and Sample Collection

**Figure 1:**
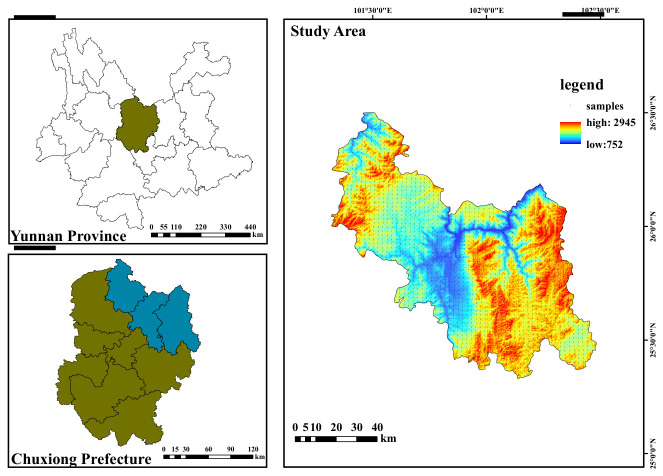
Study area.

The study area is located in the northern part of the Chuxiong Yi Autonomous Prefecture, Yunnan Province, China, with geographical coordinates ranging from 101°19′8.4″ E to 102°30′50.4″ E longitude and 25°19′40.8″ N to 26°29′56.4″ N latitude (Figure 1). It features a subtropical low-latitude plateau monsoon climate, with an average annual temperature of 17.9 °C. The region experiences large diurnal temperature variations but small annual temperature fluctuations. Rainfall and heat periods coincide, with approximately 90% of the precipitation concentrated between May and October. The average annual precipitation is 809.1 mm, while the average annual evaporation reaches 2432 mm, making it a typical arid area in central Yunnan. The terrain is higher in the center and lower in the north and south, with elevations ranging from 752 m to 2945 m. Mountainous and hilly landscapes dominate, exhibiting significant vertical climatic variation. Within Chuxiong Prefecture, cultivated land is the primary land use type, totaling 334,206.88 hectares, of which dryland accounts for 64.28%. The remaining land is predominantly used for forestry and pasture, with scattered residential areas. This diverse terrain supports a variety of agricultural activities, from lowland crops to highland livestock farming, reflecting the region’s adaptability to its natural environment.

### 2.2. Sample Collection and Analysis

During 2020–2022, a total of 1790 surface soil samples (0–20 cm depth) were collected from farmland, woodland, and orchards. Sampling avoided areas with severe soil erosion or where the topsoil had been disturbed. A kilometer-grid sampling approach was employed, with 4 km^2^ as a sampling unit and central points spaced at 2 km intervals. At each central point, one core sample and three subsamples within a 50 m radius were collected. Subsamples were homogenized in equal proportions to form a composite sample (≥1.4 kg). To ensure quality control, duplicate samples were collected by different inspectors at different times, comprising 2% of the total samples, and were inserted into each batch in a coded manner. After collection, all samples were placed in labeled polyethylene bags and naturally air-dried for one week in a ventilated indoor area before being preserved in the laboratory for subsequent analysis. During the sampling process, the accuracy of sampling points was ensured through GPS track monitoring. In the laboratory, the quality of soil sample analysis was controlled using a combination of external and internal quality control measures. Certified reference materials (GWB) were included to ensure the accuracy, compliance rate, and precision of the analytical methods.

The chromium (Cr) content was determined in accordance with Chinese Industry Standard DZ/T 0279.1-2016 Analysis methods for regional geochemical sample-Part 1: Determination of 24 components including aluminum oxide, etc., by pressed power pellets-X-ray fluorescence spectrometry [36]. Nickel (Ni), zinc (Zn), and phosphorus (P) were determined according to DZ/T 0279.2-2016 Analysis methods for regional geochemical sample-Part 2: Determination of 27 components including calcium oxide, etc., by inductively coupled plasma atomic emission spectrometry [37]. Copper (Cu) and lead (Pb) were analyzed following DZ/T 0279.3-2016 Analysis methods for regional geochemical sample-Part 3: Determination of 15 elements including barium, beryllium, bismuth, etc., by inductively coupled plasma mass spectrometry [38]. Cadmium (Cd) quantification was performed in accordance with DZ/T 0279.5-2016 Analysis methods for regional geochemical sample-Part 5: Determination of cadmium contents by inductively coupled plasma mass spectrometry [39]. Arsenic (As) was determined according to Chinese Industry Standard DZ/T 0279.13-2016 Analysis methods for regional geochemical sample-Part 13: Determination of arsenic, antimony and bismuth contents by hydride generation-atomic fluorescence spectrometry [40]. Mercury (Hg) analysis followed DZ/T 0279.17-2016 Analysis methods for regional geochemical sample-Part 17: Determination of mercury by vapor generation-cold atomic fluorescence spectrometry [41]. Nitrogen (N) content was quantified using DZ/T 0279.29-2016 Analysis methods for regional geochemical sample-Part 29: Determination of nitrogen contents by Kjeldahl distillation-volumetric method [42]. Soil pH was measured in accordance with DZ/T 0279.34-2016 Analysis methods for regional geochemical sample-Part 34: Determination of pH by ion selective electrode method [43]. Organic carbon content was determined by the potassium dichromate oxidation method with oil-bath heating.

### 2.3. Assessment Indicators for Heavy Metal Pollution

#### 2.3.1. Enrichment Factor (EF)

The Enrichment Factor (EF), initially proposed by Chester and Gladney during their investigation of lead content in atmospheric particulates [44], serves as a quantitative metric for assessing pollution levels and identifying contamination sources. This method evaluates anthropogenic impacts on heavy metal concentrations in environmental media [45]. It typically employs conservative elements characterized by crustal abundance, minimal anthropogenic influence, and geochemical stability as reference elements. In this study, titanium (Ti) was selected as the reference element. The computational methodology is detailed in Equation (1).

(1) $$EF=\frac{{\left(\frac{C_{ij}}{C_{ref}}\right)}_{sample}}{{\left(\frac{B_{i}}{B_{ref}}\right)}_{background}}$$

where (C_ij_/C_ref_)_sample_ represents the ratio of the target metal element i to the reference metal element in the sample j, and (B_i_/B_ref_)_sample_ denotes the background ratio of the target metal element to the reference metal element. This study employs the soil element background values of Yunnan Province for assessment [46]. EF values are classified into six enrichment levels: not enriched (EF < 1), mildly enriched (1 ≤ EF < 2), moderately enriched (2 ≤ EF < 5), highly enriched (5 ≤ EF < 20), intensely enriched (20 ≤ EF < 40), and very highly enriched (EF ≥ 40) [47,48].

#### 2.3.2. Geoaccumulation Index (Igeo)

The Geoaccumulation Index (I_geo_), initially proposed by Müller (1969), quantifies sediment contamination intensity through comparative analysis with pre-industrial background levels [49]. The computational methodology is presented in Equation (2).

(2) $$I_{geo}=\log_{2}[C_{ij}/(C_{ib}\times 1.5)]$$

Based on the Geoaccumulation Index, soil contamination intensity is classified into seven grades: practically unpolluted (I_geo_ < 0), unpolluted to moderately (0 ≤ I_geo_ < 1), moderately polluted (1 ≤ I_geo_ < 2), moderately to strongly (2 ≤ I_geo_ < 3), strongly polluted (3 ≤ I_geo_ < 4), strong to very strong (4 ≤ I_geo_ < 5), very strong pollution (I_geo_ ≥ 5).

#### 2.3.3. Nemerow Integrated Pollution Index (PN)

The Nemerow Integrated Pollution Index (PN) is a multi-factor environmental quality assessment method originally developed by Nemerow (1974) for water pollution evaluation, subsequently adopted extensively for soil contamination assessment [50,51,52]. Computational procedures are detailed in Equations (3) and (4).

(3) $$p_{i}=\frac{C_{ij}}{C_{ib}}$$

where P_i_ represents the Single Factor Pollution Index, C_ij_ denotes the measured concentration of the heavy metal element i in the sample j, and C_ib_ corresponds to the background value for the respective heavy metal. Based on the Single Factor Pollution Index (P_i_) values, soil pollution levels are classified into five grades: unpolluted (P_i_ < 1), mild pollution (1 ≤ P_i_ < 2), low pollution (2 ≤ P_i_ < 3), moderate pollution (3 ≤ P_i_ < 5), severe pollution (P_i_ ≥ 5).

(4) $$P_{N}=\sqrt{\frac{(P_{i,max}{)}^{2}+(P_{i,avg}{)}^{2}}{2}}$$

where P_N_ represents the Nemerow Integrated Pollution Index, P_i,max_ denotes the maximum measured concentration of the same element, and P_i,avg_ signifies the arithmetic mean concentration of the same element. Soil pollution levels are classified into five grades based on P_N_ values: unpolluted (P_N_ < 0.7), mild pollution (0.7 ≤ P_N_ < 1), low pollution (1 ≤ P_N_ < 2), moderate pollution (2 ≤ P_N_ < 3), severe pollution (P_N_ ≥ 3).

#### 2.3.4. Potential Ecological Risk Index (RI)

The Potential Ecological Risk Index (RI) was proposed by Hakanson in 1980, based on the theory of aquatic sedimentology [53]. This index quantifies the potential threat of pollution to ecosystems by incorporating heavy metal concentrations, toxicity levels, environmental sensitivity, and pollutant types. It has subsequently been extended to assess pollution in soils, the atmosphere, and other environmental media. The specific calculation method is presented in Equation (5).

(5) $$RI=\sum E_{i}=\sum (T_{i}\times C_{ij})=\sum (T_{i}\times C_{ij}/C_{ib})$$

where E_i_ represents the Environmental Risk Index for heavy metals, T_i_ denotes the toxicity response coefficient for heavy metals (Hg = 40, Cd = 30, Pb = 5, As = 10, Cr = 2, Cu = 5, Ni = 5, Zn = 1). Based on the RI value, the degree of soil ecological risk can be classified into four levels: low risk (RI < 150), moderate risk (150 ≤ RI < 300), high risk (300 ≤ RI < 600), and very high risk (RI ≥ 600).

### 2.4. Methods for Source Apportionment of Heavy Metals in Soils

#### 2.4.1. Absolute Principal Component Scores-Multiple Linear Regression (APCS-MLR)

The APCS-MLR model quantifies the contribution rates of individual pollution sources through multivariate linear regression based on Principal Component Analysis. This approach shows enhanced flexibility and computational efficiency compared to conventional receptor modeling methods [54]. The specific computational procedures are detailed in Equations (6)–(8).

(6) $$Z_{ij}=\frac{C_{ij}-C_{avg}}{\sigma_{i}}$$

(7) $${\left(z_{0}\right)}_{i}=\frac{0-C_{i,avg}}{\sigma_{i}}=-\frac{C_{i,avg}}{\sigma_{i}}$$

(8) $$X_{i}=b_{0}+\sum_{k=1}^{m}b_{k}\times APCS_{k}$$

where Z_ij_ denotes the standardized concentration of heavy metal i in sample j, C_ij_ represents the measured concentration of heavy metal i in sample j. C_avg_ is the mean concentration of heavy metals across all samples. σ_i_ signifies the standard deviation of heavy metal elements. b_k_ indicates the regression coefficient of heavy metal elements. m corresponds to the number of pollution sources. APCS_k_ refers to the source-adjusted absolute factor score for source k.

#### 2.4.2. Positive Matrix Factorization (PMF)

The EPA PMF 5.0 model, developed by the United States Environmental Protection Agency (US EPA), is a multivariate factor analysis tool. It employs weighted least-squares minimization to decompose the original data matrix into factor contribution matrices and factor profile matrices [55]. This approach enables robust apportionment of pollution sources. The calculation method is shown in Equations (9)–(11).

(9) $$x_{ij}=\sum_{k=1}^{p}g_{jk}\times f_{ki}+e_{ij}$$

x_ij_ denotes the concentration of heavy metal i in sample j. g_jk_ represents the contribution of pollution source k to sample j. f_ki_ signifies the compositional profile of heavy metal i in source k. e_ij_ indicates the residual error for metal i in sample j.

(10) $$Q={\sum_{i=1}^{n}\sum_{j=1}^{m}\left[\frac{x_{ij}-\sum_{k=1}^{p}g_{jk}\times f_{ki}}{u_{ij}}\right]}^{2}$$

where u_ij_ represents the measurement uncertainty of heavy metal i in sample j.

(11) $$U_{ij}=\left\{\begin{array}{c} \left(\frac{5}{6}\right)\times MDL,\;c_{ij}\;\leq \;MDL\\ \sqrt{{\left(\sigma \times c_{ij}\right)}^{2}+{\left(0.5\times MDL\right)}^{2}} \end{array}\right.$$

σ denotes the error fraction, C_ij_ represents the measured concentration of heavy metal i in sample j. MDL signifies the method detection limit for heavy metal elements. Concentrations below the detection limit are calculated according to Equation (12) [56]:

(12) $$x_{ij}=\frac{MDL}{2},\sigma_{ij}=\frac{5MDL}{6}$$

For concentrations exceeding the detection limit, the uncertainty is calculated according to Equations (13) and (14):

(13) $$\sigma_{ij}=\frac{MDL}{3}+0.2\times c_{ij},x_{ij}\leq 3MDL$$

(14) $$\sigma_{ij}=\frac{MDL}{3}+0.1\times c_{ij},x_{ij}>3MDL$$

#### 2.4.3. Random Forest

Random Forest, a Bootstrap Aggregating (Bagging) ensemble method, reduces variance and mitigates overfitting risk by training multiple decision trees in parallel and aggregating their predictions. It is characterized by both sample randomness and feature randomness. The model constructs regression trees using bootstrap samples (random sampling with replacement) from the dataset. The final prediction for the dependent variable is determined by averaging the outputs of the individual regression trees. To reduce inter-feature correlations, the data is randomly partitioned into a training set for model calibration and a test set for model evaluation [57]. Model performance can be optimized during training by tuning key hyperparameters, including the number of trees, the number of candidate features considered at each split (mtry), and the minimum node size [58].

### 2.5. Statistical Analysis

Data preprocessing was performed using Excel 2021. Graphical representations were generated with Origin 2022 and spatial distribution maps were created using ArcGIS 10.8. Spatial modeling and prediction of the random field were conducted via the Kriging method based on covariance functions. Positive Matrix Factorization (PMF) analysis was implemented using EPA PMF 5.0, while Absolute Principal Component Score (APCS) linear regression analysis was performed with SPSS 27.0.1. Random Forest modeling was executed in R 4.4.2. Heavy metal sources in soils were subsequently determined by synthesizing the results from the above analyses.

## 3. Results

### 3.1. Characteristics of Soil Heavy Metal Concentrations in the Study Area

#### 3.1.1. Descriptive Statistics

Table 1 presents the statistical summary of concentrations for eight heavy metals (Hg, Cd, Pb, As, Cr, Cu, Ni, and Zn) in soils within the study area. The mean concentrations of Hg, Cd, Pb, As, Cr, Cu, Ni, and Zn were 0.03, 0.17, 25.01, 7.46, 85.91, 36.20, 31.75, and 69.24 mg/kg, respectively. Among these, the mean concentrations of all seven heavy metals except Cr were lower than the soil background values for Yunnan Province. The proportions of sampling sites exceeding Yunnan Province’s background values for each of the eight heavy metals were 5.70%, 18.10%, 4.75%, 2.68%, 87.54%, 11.01%, 10.45%, and 12.63%, respectively. Furthermore, the proportions exceeding soil screening values were all below 2%. Within the study area, soil Hg, Cd, Pb, As, and Cd concentrations were lower than the risk intervention thresholds stipulated by China’s soil environmental quality risk control standard for soil contamination of agricultural land (GB 15618-2018) [59]. The standards do not specify thresholds for Cu, Ni, and Zn. The coefficient of variation (CV) indicates the degree of influence from external sources on heavy metal concentrations in soils. Higher CV values suggest greater anthropogenic or external impacts. According to conventional classification, CV values are categorized into three levels: low variation (0–15%), moderate variation (15–35%), and high variation (>35%) [60]. In the study area, the coefficients of variation (CV) for all eight soil heavy metals, except Cr, exceeded 35%, indicating high variation. This suggests relatively strong external influences on their sources. Collectively, these results demonstrate generally favorable soil quality across the region. However, localized areas exhibit significant heavy metal enrichment, presenting elevated risks of point-source pollution that merit further investigation into their origins.

#### 3.1.2. Spatial Distribution of Heavy Metals

**Figure 2:**
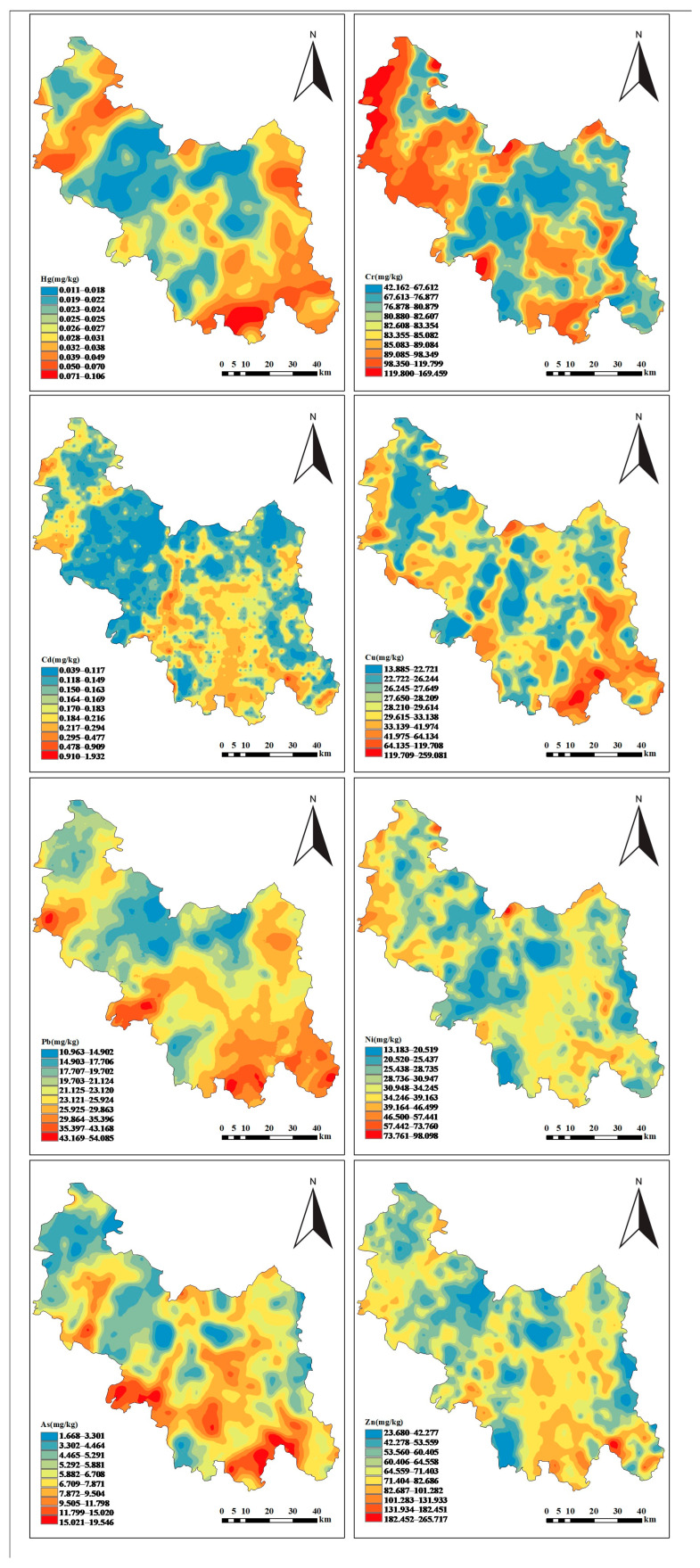
Spatial distribution of soil heavy metal concentrations.

Figure 2 illustrates the spatial distribution of heavy metal concentrations across the study area. The Jinsha River Basin in the area follows a north–south structural trend, with tributaries exhibiting a fan-shaped distribution extending from the northeast. The distribution of all heavy metal elements showed similar patterns.

Within the study area, Cd, As, Cu, Ni, and Zn exhibit spatially extensive distributions. Their high-concentration zones predominantly demonstrate linear patterns that broadly align with regional hydrological networks, exhibiting outward dispersion trends. In contrast, Hg, Pb, and Cr display more localized distributions, with high-concentration zones primarily characterized by areal clustering patterns concentrated in the eastern and western sectors of the region. The high-concentration zones of all eight heavy metals exhibit significant spatial overlap. Within the southern sector of the study area, these zones display a characteristic pattern of radiation outward from river channels. Hydrological dynamics, particularly the flow directions of rivers and their tributaries, govern heavy metals’ migration. This fluvial influence is most pronounced in the southern river network corridors.

Soil pH within the study area exhibits a pronounced north–south dichotomy, with acidic soils dominating the southern sector and alkaline soils prevailing in the northern sector. This marked pH gradient contributes significantly to the spatial heterogeneity of Hg, Cd, Pb, and Cr, generating complex distribution patterns. In Yunnan’s characteristic karst terrain, heavy metals inputs demonstrate seasonal source alternation: natural sources dominate during the wet season, while anthropogenic sources prevail in the dry season [61]. Based on the dual sources of regional heavy metals and the regulatory influence of surface water on their migration in soil, the complexity of regional environmental remediation is further intensified. Concurrently, under the combined effects of topography and hydrological flow, soil heavy metals concentrations in downstream river areas and river valleys are often notably elevated relative to other regions [62,63]. Based on the above research, significant spatial heterogeneity in soil heavy metal concentrations has been identified across the study area.

### 3.2. Regional Soil Quality Assessment

#### 3.2.1. Heavy Metal Pollution Assessment

**Figure 3:**
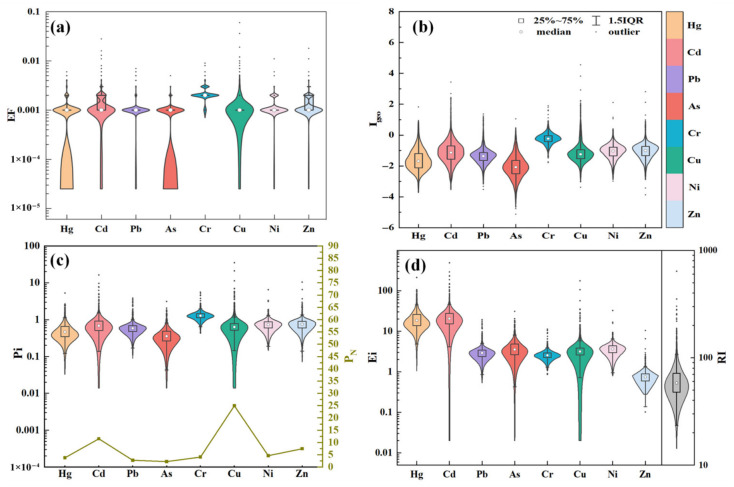
Soil heavy metal indices in the study area: EF (**a**), Igeo (**b**), P_N_ (**c**), RI (**d**).

The statistical results of EF, Igeo, and PN for assessing heavy metal pollution levels in the study area are presented in Table 2, Figure 3a–c, respectively. The results demonstrate that the EF for all eight heavy metals at all sites within the study area are less than 1, indicating the absence of significant enrichment.

The mean Geoaccumulation Index values for all eight heavy metals were negative, with the following order: Cr (−0.23) > Zn (−1.05) > Ni (−1.08) > Cd (−1.13) > Cu (−1.17) > Pb (−1.38) > Hg (−1.62) > As (−2.07). The proportion of sampling points reaching moderately polluted level or above is Cu (0.95%), Cd (0.73%), Cr (0.34%), Pb (0.22%), Zn (0.17%), Ni (0.17%), As (0.06%), Hg (0.06%). The statistical analysis reveals that a limited number of sampling sites reached strongly polluted levels, specifically Cd (0.06%) and Cu (0.06%). This indicates that while the overall heavy metal pollution status remains relatively low across the study area, localized contamination hotspots exist, primarily driven by Cd and Cu.

The Nemerow Integrated Pollution Index (PN) results further elucidate the overall status of soil heavy metal contamination. The proportions of sampling sites where Single Factor Pollution Index Pi reached moderate or higher pollution levels are quantified as follows: Cu (0.95%), Cd (0.73%), Cr (0.34%), Pb (0.22%), Ni (0.17%), Zn (0.17%), As (0.06%), Hg (0.06%). Based on the mean values of Single Factor Pollution Index Pi, the heavy metals are ranked in ascending order as follows: Cr (1.32) > Cd (0.79) > Cu (0.78) > Zn (0.77) > Ni (0.75) > Pb (0.62) > Hg (0.55) > As (0.41). Based on the PN, the heavy metals are ranked in descending order of contamination severity: Cu (24.95) > Cd (11.50) > Zn (7.45) > Ni (4.62) > Cr (4.07) > Hg (3.77) > Pb (2.77) > As (2.22). All elements except Pb and As exhibited severe pollution levels (PN ≥ 3), while Cu displayed the most catastrophic contamination level (PN = 24.95).

The comprehensive index assessments collectively indicate that the overall soil quality in the study area remains acceptable, with heavy metal contamination generally within manageable levels. However, sporadic contamination hotspots exhibit anomalously elevated concentrations, necessitating targeted focus on high-concentration distribution zones of Cu, Cd, and Cr. Implementing effective risk mitigation strategies is critical to prevent further dispersion and environmental degradation.

#### 3.2.2. Potential Ecological Risk Assessment

As shown in Figure 3d, the Potential Ecological Risk Index (RI) reveals the ecological hazards of heavy metals in the study area. The single environmental risk factors were ranked by descending mean values: Cd (23.56) > Hg (21.88) > As (4.05) > Cu (3.91) > Ni (3.74) > Pb (3.08) > Cr (2.64) > Zn (0.77). The study area demonstrates predominantly low ecological risk, with 1762 sampling sites (98.44%) registering low risk levels (RI < 150). Moderate risk (150 ≤ RI < 300) occurs at 25 sites (1.40%), while significantly elevated risk manifests at high-risk locations (300 ≤ RI < 600, two sites, 0.11%) and very-high-risk sites (RI ≥ 600, one site, 0.06%). These findings indicate predominantly low potential ecological risks from heavy metals across the study area, though discernibly higher risks manifest in localized zones, with Cd and Hg identified as primary contributing factors.

### 3.3. Sources of Soil Heavy Metals

#### 3.3.1. Main Factors Influencing Heavy Metals Accumulation in Soil

**Figure 4:**
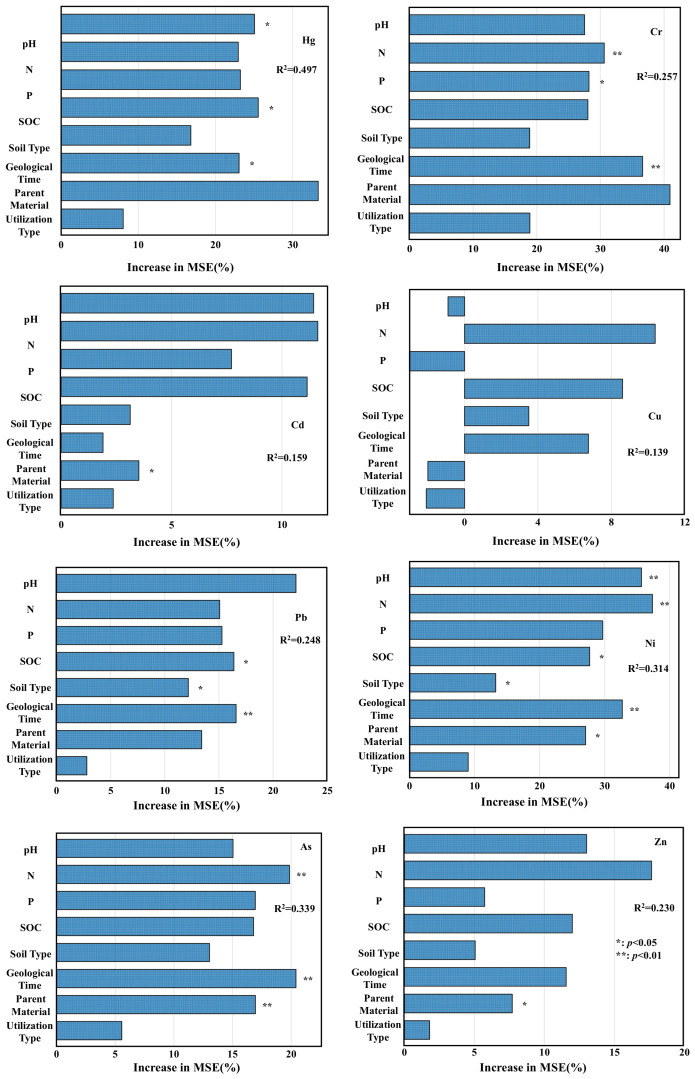
Factors influencing concentrations of eight heavy metals in soils.

Random Forest models effectively reveal the multivariate sources of heavy metals in soils, complementing results from Positive Matrix Factorization (PMF) and Absolute Principal Component Score-Multiple Linear Regression (APCS-MLR) analyses. This study employed Random Forest modeling to analyze the impacts of soil pH, phosphorus content, nitrogen content, organic carbon content, soil type, geological time, parent material, and land utilization type on the concentrations of eight heavy metals in regional soils. Bayesian optimization was employed to predict Random Forest model performance, identify optimal hyperparameters, and evaluate model reliability, with results summarized in Table 3. Through cross-validation, the associations between key factors and the eight heavy metals were further validated, ensuring precision in source apportionment. Considering significant predictors and variable importance, the analytical results of factor-heavy metal relationships are visualized in Figure 4. Based on a 15% change in Mean Squared Error (MSE) of perturbed soil heavy metal concentrations as the importance criterion, soil pH, N, P, SOC, geological time, and parent material primarily influence Hg, Cr, and Ni. Soil pH and organic carbon content dominate Hg accumulation; soil pH, N, and organic carbon content governs Cd distribution; geological time and parent material control As and Pb; geological time combined with nitrogen phosphorus inputs jointly regulate Cr, Cu, and Ni, while soil pH and organic carbon govern Zn. The interactions among these factors further elucidate the complexity of regional heavy metal distribution patterns.

Based on Random Forest modeling results, soil heavy metal sources can be categorized into natural and anthropogenic origins. The accumulation of Hg, Cd, Pb, As, and Cu exhibits significant compound influences from both sources, while Cr, Ni, and Zn demonstrate analogous trends in inter-group analysis, though no single dominant factor exclusively governs their accumulation patterns.

#### 3.3.2. Source Apportionment via APCS-MLR

The standardized dataset underwent Kaiser-Meyer-Olkin (KMO) and Bartlett’s sphericity tests, yielding values of 0.652 and <0.001, respectively, confirming suitability for principal component analysis (PCA). PCA extracted three principal components (PCs) that cumulatively explained 68.68% of the total variance in eight heavy metals. The variance contributions were as follows: PC1 (36.41%), PC2 (19.28%), and PC3 (12.98%), with detailed loadings presented in Table 4. Principal component 1 (PC1) exhibited high loadings for Zn (0.87), Cd (0.82) and Pb (0.67); PC2 showed elevated loadings for Cr (0.93) and Ni (0.90); PC3 was dominated by Cu (0.82), Hg (0.61) and As (0.51).

**Figure 5:**
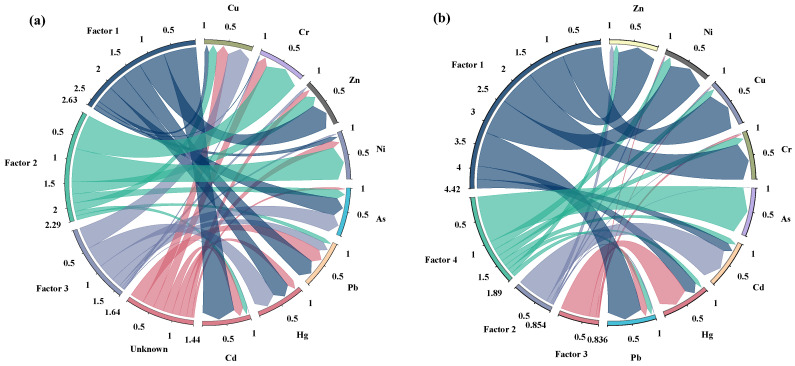
Source factor contributions from APCS-MLR (**a**) and PMF (**b**) analyses.

To further clarify the sources and quantitative contributions of eight heavy metals in the study area, the APCS-MLR model was employed to analyze measured heavy metal concentrations. The results demonstrate adjusted R^2^ values ranging from 0.55 to 0.95, indicating satisfactory model fit. For all eight heavy metals, *p*-values from the F-test were below 0.01, confirming statistically significant correlations between soil metals and their identified sources. Source apportionment analysis resolved three identified sources and one unknown source, with relative contributions of 32.87%, 28.64%, 20.51%, and 17.98%, respectively. The contribution magnitude of each factor to soil heavy metals is visualized in Figure 5a. Factor 1 exhibited significant contributions (>20%) to Cd (69.72%), Zn (56.49%), Pb (41.37%), Hg (38.09), and As (37.77%). Factor 2 dominated (>20%) Cr (75.15%), Ni (71.19%), and Zn (26.35%). Factor 3 primarily contributed to Cu (47.29%), Hg (45.93%), As (38.06%), and Pb (20.13%), with all other elements showing contributions below 20%. The unknown factor exhibited substantial contributions (>20%) to Pb (29.57%), Cu (24.92%), and Cr (22.05%), while contributions to all other elements remained below 20%. Comparative analysis indicated that Hg, Pb, Cu and Zn showed significant contributions across multiple factors, indicating diverse sources and complex enrichment pathways.

#### 3.3.3. Source Apportionment via PMF

This study employed the Positive Matrix Factorization (PMF) model for quantitative source apportionment of soil heavy metals, while simultaneously validating the accuracy of the APCS-MLR model. Analysis was performed using EPA PMF 5.0 software, with 100 bootstrap runs conducted for factor numbers ranging from 3 to 6. The consistent Qrobust/Qtrue ratio of 1.0 across all models indicates minimal influence of data outliers on the model results. As the factor number increased, the Q value progressively stabilized and model fit improved, albeit with rising overfitting risks. At four factors, a marked decrease in Q was observed, indicating that this configuration optimally resolves heavy metal source characteristics. The contribution rates of each factor to soil heavy metals are presented in Figure 5b. PMF modeling supported four factors with relative contributions of 55.21%, 10.67%, 10.45%, and 23.66%, respectively. Factor 1 was primarily associated with Cu (83.74%), Zn (80.19%), Cr (78.69%), Ni (77.72%), and Pb (73.51%), Factor 2 dominated Cd (69.18%), Factor 3 showed the strongest association with Hg (62.36%), while Factor 4 predominantly influenced As (99.99%).

Comparative analysis of APCS-MLR and PMF results indicated high consistency between both models in resolving soil heavy metal sources. Based on comparative model performance, A-M1 (Absolute principal component linear regression analytical factor 1) and PMF1 (Positive Matrix Factorization analysis Factor 1) are identified as source 1, while A-M2 and PMF4 form source 2. Source 3 consists of A-M3 and PMF3, with A-M Unknown and PMF2 constituting source 4. Source 1 is primarily associated with Cd, Pb, As, Cu, Ni, and Zn. According to publicly available reports, the study area has a long history of limestone mining activities, which explains its classification as an industrial hybrid source. Source 2 mainly relates to Cr, Ni, and As, which are closely associated with parent material and geogenic background in many studies [64,65]. Therefore, source 2 is classified as a natural background source. Source 3 involves Hg and Pb, frequently associated with atmospheric transport and deposition processes, identified as atmospheric deposition sources [66]. Given that Cd is commonly influenced by agricultural practices such as fertilizer and agrochemical application, this factor was classified as an agricultural source. The dominant sources are industrial hybrid sources (32.87%, 55.21%) and natural background sources (28.64%, 23.66%).

## 4. Discussion

### 4.1. Cross-Validation of Pollution Assessment Results and Ecological Implications

Comparing pollution classification results from the Enrichment Factor (EF) and Geoaccumulation Index (Igeo) supported high consistency between these assessment methods. This agreement not only shows their strong indicative capacity for pollution evaluation but also empirically corroborates their reliability. Tiered classification based on mean values of the Enrichment Factor (EF) and Geoaccumulation Index (Igeo) consistently identified Cr as exhibiting significantly higher enrichment levels than other elements. As both indices are calculated from single-element concentrations relative to background values, this pattern strongly indicates substantial anthropogenic influence on Cr accumulation [67]. Simultaneously, Cd, Zn, and Ni exhibited elevated pollution evaluation values, indicating substantial anthropogenic influences on their accumulation in soils. Regions with higher contamination levels displayed largely consistent spatial distributions and concentration characteristics, revealing a pattern of discrete hotspots superimposed on diffuse contamination. This spatial congruence proves the complexity and multiplicity of their emission pathways.

In numerous studies on the characteristics of soil pollution in river valleys and arid regions, indices such as EF and Igeo are widely used to represent the characteristics of soil heavy metal pollution [68,69,70]. However, in the process of conducting more in-depth investigations into the underlying mechanisms, these indices still exhibit certain limitations. Additionally, their calculation process is heavily dependent on reference elements. Considering the influence of different regional characteristics and human activities, it is important not to overly rely on the performance of EF and Igeo when evaluating soil pollution. A more comprehensive and systematic evaluation approach should be combined for a more thorough investigation.

Analysis using the Nemerow Integrated Pollution Index (PN) provides comprehensive assessment of regional heavy metal contamination levels, while Single Factor Pollution Index (Pi) characterizes site-specific elemental pollution. Their integration enables a multi-angled evaluation of soil quality across the study area. For the Single Factor Pollution Index (Pi), Cu exhibited the highest proportion of sites reaching moderate pollution levels, albeit at a minimal 0.95%. In contrast, the Nemerow Integrated Pollution Index (PN) demonstrated that all heavy metals collectively attained moderate pollution status across the study area, with Cu, Cd, and Zn being particularly pronounced. Due to their distinct computational emphases, the Single Factor Index (Pi) better characterizes the spatial heterogeneity of heavy metals, while the Nemerow Integrated Pollution Index (PN) integrates mean and extreme values to assess regional soil quality holistically. However, in areas with pronounced spatial variability, PN’s susceptibility to extreme values may compromise assessment accuracy. Both the individual Environmental Risk Index (Ei) and the Potential Ecological Risk Index (RI) are calculated based on site-specific heavy metal concentrations, with Cd and Hg identified as the most prominent risk drivers. In summary, the Enrichment Factor (EF), Geoaccumulation Index (Igeo), Single Factor Pollution Index and individual Environmental Risk Index indicates the spatial distribution patterns of soil quality within the study area. Meanwhile, the Nemerow Integrated Pollution Index and the Potential Ecological Risk Index provide a robust assessment of the overall soil quality level across the region. Notably, both categories of indices demonstrate high consistency in their findings within this study. Cd and Hg are highly toxic heavy metals that, after entering the environment through both natural and anthropogenic activities, can accumulate in ecosystems over long periods and magnify along the food chain. Once Cd enters the human body, it interferes with calcium metabolism, leading to osteoporosis and renal tubular dysfunction. The oxidative stress caused by Cd in the body can also induce DNA damage. Hg primarily affects the nervous and reproductive systems. In particular, methylmercury can cross the blood–brain barrier and accumulate in the central nervous system, causing ataxia, speech disorders, and cognitive decline. Therefore, for areas severely contaminated by Cd and Hg, comprehensive measures such as long-term ecological monitoring and source control should be implemented alongside pollution remediation to mitigate ecological risks.

Through the calculation of the Enrichment Factor (EF), Geoaccumulation Index (Igeo) and Nemerow Integrated Pollution Index (PN), the soil quality within the study area can be quantitatively characterized and assessed from multiple perspectives. Meanwhile, the Potential Ecological Risk Index (RI) effectively reflects the ecological effects of heavy metals in the soil. The combined application of these indices enables the accurate identification of high-risk heavy metal elements within the region. Notably, the RI values for Cd and Hg in the study area strongly exceeded those of other elements. Elevated RI values for specific elements are primarily attributed to their high accumulation levels in the soil and inherent high toxicity.

### 4.2. Joint Validation of APCS-MLR and PMF Models and Analysis of Driving Mechanisms

The analysis results from both models indicate that in identifying the sources of heavy metal elements, the APCS-MLR model indicated a greater number of distinct contribution factors. In contrast, the PMF model demonstrated a stronger focus on contributions from individual sources. This discrepancy arises because the orthogonality constraint and linear assumptions inherent in APCS-MLR impair its ability to effectively resolve composite sources. Conversely, the non-negative decomposition and error weighting scheme employed by the PMF model enable it to more effectively resolve mixed sources [71]. This also indicates that while the APCS-MLR model can identify a greater number of potential sources when dealing with elements influenced by complex interactions, the accuracy of its source resolution may be compromised. Conversely, although the PMF model exhibits higher sensitivity to contributions from individual sources, it shows superior capability in resolving multi-source mixtures. In studies related to mining sites, PMF identified that heavy metals like Pb, Cd, Cu, and Zn were predominantly influenced by smelter activities (slags, wastewater discharge), while metals such as Cr and Mn were attributed to natural sources [72]. A study on agricultural soil pollution source apportionment across multiple regions in South Korea attributed As, Cd, Cr, and Ni to anthropogenic pollution sources, while attributing the contamination of Cu, Hg, Pb, and Zn to the combined effects of anthropogenic and natural sources [73]. The observed discrepancies in results are largely attributable to variations in land use patterns across regions. For diverse ecosystems such as agricultural and industrial zones, adequate and precise sample sizes are essential to ensure the accuracy of pollution source APCS-MLR and PMF analyses during source apportionment [74,75]. In practical applications, the integrated application of both models leverages their respective strengths, enabling a more refined and comprehensive source apportionment of soil heavy metal contamination. This integrated approach provides a scientific basis for guiding the remediation of contaminated soils [76].

The concentration of heavy metals in soil is influenced by multiple factors, including, but not limited to, soil type, pedogenesis (soil evolution), and anthropogenic activities, representing the collective influence of both natural and anthropogenic processes [77]. The complex interplay of these factors shapes the spatial distribution patterns of heavy metal concentrations in soils [78]. The parent material determines the background levels of heavy metals, with their initial release occurring through bedrock weathering. Particularly in karst terrain, variations in parent materials exert a pronounced influence on the distribution of heavy metals in soils. The elements most notably influenced by this are Hg, Cd, Pb, Ni, and Zn [79]. Soil pH and soil type ultimately determine heavy metal concentrations in soils by governing two key processes: their bioavailability and their migration/accumulation behavior. Specifically, pH significantly influences the activity and solubility of heavy metals, typically exhibiting a negative correlation with pH [80]. Conversely, the properties of the parent material and clay minerals govern the adsorption capacity for heavy metals within the soil matrix [81]. Furthermore, climatic conditions, such as precipitation and temperature, also influence the migration and transformation of heavy metals in soils [82]. Precipitation alters the pH of surface soil layers and directly or indirectly affects their accumulation processes through hydraulic transport, with Cd, As, and Cr being the most significantly impacted elements [83]. Vegetation type and associated root activity similarly play critical roles in the immobilization and release of heavy metals. Root exudates modify the soil microenvironment, thereby influencing heavy metal bioavailability [84].

**Figure 6:**
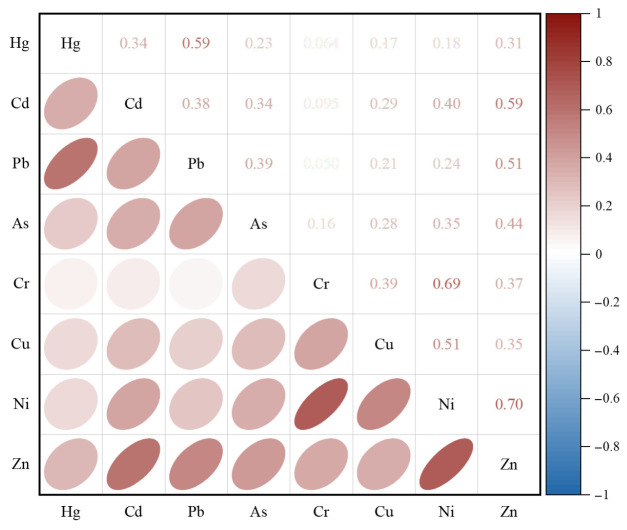
Correlation analysis of various heavy metals.

Simultaneously, human activities significantly influence heavy metal migration and accumulation through pathways such as altering soil properties and increasing external inputs. In this study, Hg, Cr, Pb, and Zn exhibited significant correlations with natural and anthropogenic factors including soil N and P concentrations and parent material. Correlation analysis (Figure 6) further indicated common sources among these heavy metal elements. This indicates that, within the study area, elevated geogenic background levels contribute to heavy metal presence, with natural weathering processes promoting their release [85]. Anthropogenic activities, notably agricultural fertilization and mining operations, have significantly enhanced the migration and accumulation of these heavy metals in the soil [86,87]. In agricultural soils under long-term cultivation, while fertilizer application and straw returning may marginally elevate heavy metal contents, the predominant enrichment is primarily attributed to atmospheric deposition [88]. Current research commonly attributes elevated Cr concentrations primarily to local background levels, whereas the enrichment of Hg, Pb, and As is predominantly influenced by anthropogenic activities [89]. This spatial pattern is further evidenced by the predominant distribution of their high-value zones within industrial clusters and high-traffic areas of the study region.

## 5. Conclusions

The study indicated elevated exceedance rates for Cr, Cd, Zn, Cu, and Ni. Furthermore, heavy metal concentrations exhibited significant geographical heterogeneity, with high-value zones demonstrating a distinct mixed linear and areal distribution pattern. Enrichment Factor (EF) and Geoaccumulation Index (Igeo) indicated slight contamination by Cr, Cd, and Cu. In contrast, the Nemerow Integrated Pollution Index (PN) and Potential Ecological Risk Index (RI) identified Hg, Cd, and Cu as predominant contaminants, with Cd and Hg posing substantial environmental and ecological risks. Utilizing the Random Forest model to assess the influence of environmental factors on eight heavy metals indicated that the accumulation of Hg, Pb, As, Cr, Ni, and Zn in soils is subject to significant combined influences from both natural and anthropogenic sources. Key contributing factors include geological time, parent material, pH, N, and P levels. Four primary heavy metal sources were apportioned using APCS-MLR and PMF: industrial hybrid sources (32.87%, 55.21%), natural background sources (28.64%, 23.66%), atmospheric deposition sources (20.51%, 10.45%), and agricultural sources (17.98%, 10.67%). Among these, the industrial hybrid sources and natural background sources were identified as the predominant contributors.

The results obtained from APCS-MLR and PMF exhibited broad consistency. Their combined application proved effective in resolving heavy metal sources in agricultural soils. For geogenic high-background regions like the Yunnan Province, enhanced long-term monitoring of agricultural soils and the implementation of preventative management measures against heavy metal pollution are recommended. These findings have significant scientific and practical value for subsequent regional pollution control and ecological restoration, especially in karst regions and similar areas.

## Figures and Tables

**Table 1:** Soil heavy metal concentration characteristics.

	Hg	Cd	Pb	As	Cr	Cu	Ni	Zn
Maximum (mg/kg)	0.31	3.54	157.00	57.20	365.00	1633.00	276.00	942.00
Minimum (mg/kg)	0.01	0.03	5.30	0.79	28.40	6.67	8.07	9.20
Average (mg/kg)	0.03	0.17	25.01	7.46	85.91	36.20	31.75	69.24
Standard deviation	0.02	0.14	11.03	4.29	22.77	51.46	11.69	34.11
Coefficient of variation (%)	58.92	82.42	44.09	57.58	26.51	142.15	36.81	49.26
Background value of soil in Yunnan Province (mg/kg)	0.06	0.22	40.60	18.40	65.20	46.30	42.50	89.70
Proportion higher than the background value of soil in Yunnan Province (%)	5.70	18.10	4.75	2.68	87.54	11.01	10.45	12.63
Screening value of agricultural land (mg/kg)	0.50	0.40	100.00	30.00	250.00	150.00	70.00	200.00
Proportion higher than agricultural land screening value (%)	0	1.96	0.39	0.39	0.22	0.95	0.56	0.56
Agricultural land control value (mg/kg)	2.50	2.00	500.00	150.00	850.00	-	-	-
Proportion higher than Agricultural land control value (%)	0	0	0	0	0	-	-	-

“-”: Not available.

**Table 2:** Soil heavy metal pollution assessment characteristics.

	Hg	Cd	Pb	As	Cr	Cu	Ni	Zn
Mean of EF	0.001	0.001	0.001	0.001	0.002	0.001	0.001	0.001
Mean of Igeo	−1.62	−1.13	−1.38	−2.07	−0.23	−1.17	−1.08	−1.05
0 ≤ Igeo < 1	1.68%	3.52%	1.12%	0.39%	21.06%	4.47%	0.50%	1.17%
1 ≤ Igeo < 2	0.06%	0.45%	0.22%	0.06%	0.34%	0.67%	0.11%	0.06%
2 ≤ Igeo < 3	0.00%	0.22%	0.00%	0.00%	0.00%	0.17%	0.06%	0.11%
3 ≤ Igeo < 4	0.00%	0.06%	0.00%	0.00%	0.00%	0.06%	0.00%	0.00%
4 ≤ Igeo < 5	0.00%	0.00%	0.00%	0.00%	0.00%	0.06%	0.00%	0.00%
Igeo ≥ 5	0.00%	0.00%	0.00%	0.00%	0.00%	0.00%	0.00%	0.00%
Mean of Pi	0.55	0.79	0.62	0.41	1.32	0.78	0.75	0.77
Pi < 1	0.06%	0.73%	0.22%	0.06%	0.34%	0.95%	0.17%	0.17%
1 ≤ Pi < 2	6.15%	17.43%	4.19%	2.57%	84.97%	8.21%	10.56%	11.90%
2 ≤ Pi < 3	0.73%	0.84%	0.34%	0.06%	2.29%	1.84%	0.00%	0.67%
3 ≤ Pi < 5	0.00%	0.39%	0.22%	0.06%	0.22%	0.45%	0.11%	0.06%
Pi ≥ 5	0.06%	0.34%	0.00%	0.00%	0.11%	0.50%	0.06%	0.11%
PN	3.77	11.50	2.77	2.22	4.07	24.95	4.62	7.45

**Table 3:** Optimized Random Forest parameters via Bayesian optimization.

Dependent Variable	Optimal Model Parameters			Predicted R^2^
	Mtry	Nodesize	Ntree	
Hg	1	7	1461	0.781
Cd	1	15	1082	0.476
Pb	1	1	1131	0.784
As	2	2	302	0.891
Cr	3	2	1770	0.886
Cu	2	1	1837	0.875
Ni	2	13	954	0.665
Zn	2	16	1035	0.589

**Table 4:** Principal component analysis results.

Total Variance Explained									
Component	Initial Eigenvalues			Extraction Sums of Squared Loadings			Rotation Sums of Squared Loadings		
	Total	Variance/%	Cumulative/%	Total	Variance/%	Cumulative/%	Total	Variance/%	Cumulative/%
1	2.91	36.41	36.41	2.91	36.41	36.41	2.22	27.69	27.69
2	1.54	19.28	55.69	1.54	19.28	55.69	1.78	22.18	49.87
3	1.04	12.98	68.68	1.04	12.98	68.68	1.51	18.81	68.68
4	0.81	10.12	78.80	-	-	-	-	-	-
5	0.73	9.13	87.93	-	-	-	-	-	-
6	0.48	5.99	93.92	-	-	-	-	-	-
7	0.29	3.57	97.48	-	-	-	-	-	-
8	0.20	2.52	100.00	-	-	-	-	-	-

“-”: Not available.

## Data Availability

The original contributions presented in this study are included in this article. Further inquiries can be directed to the corresponding authors.
